# The mPOWERED Electronic Learning System for Intimate Partner Violence Education: Mixed Methods Usability Study

**DOI:** 10.2196/15828

**Published:** 2020-01-03

**Authors:** Charmayne Hughes, Elaine A Musselman, Lilia Walsh, Tatiana Mariscal, Sam Warner, Amy Hintze, Neela Rashidi, Chloe Gordon-Murer, Tiana Tanha, Fahrial Licudo, Rachel Ng, Jenna Tran

**Affiliations:** 1 Health Equity Institute San Francisco State University San Francisco, CA United States; 2 School of Nursing San Francisco State University San Francisco, CA United States

**Keywords:** intimate partner violence, domestic violence, nursing education, learning

## Abstract

**Background:**

Nurse practitioners are a common resource for victims of intimate partner violence (IPV) presenting to health care settings. However, they often have inadequate knowledge about IPV and lack self-efficacy and confidence to be able to screen for IPV and communicate effectively with patients.

**Objective:**

The aim of this study was to develop and test the usability of a blended learning system aimed at educating nurse practitioner students on topics related to IPV (ie, the mPOWERED system [Health Equity Institute]).

**Methods:**

Development of the mPOWERED system involved usability testing with 7 nurse educators (NEs) and 18 nurse practitioner students. Users were asked to complete usability testing using a speak-aloud procedure and then complete a satisfaction and usability questionnaire.

**Results:**

Overall, the mPOWERED system was deemed to have high usability and was positively evaluated by both NEs and nurse practitioner students. Respondents provided critical feedback that will be used to improve the system.

**Conclusions:**

By including target end users in the design and evaluation of the mPOWERED system, we have developed a blended IPV learning system that can easily be integrated into health care education. Larger-scale evaluation of the pedagogical impact of this system is underway.

## Introduction

### Background

Violence in interpersonal relationships is a substantial health and social problem in the United States, with approximately 1 in 4 women and 1 in 7 men in the United States reporting being a victim of intimate partner violence (IPV) at least once in their lifetime [1]. This is particularly concerning given that IPV victimization often results in adverse psychological problems (eg, posttraumatic stress, depression, and low self-esteem) [2]; harmful health behaviors (eg, substance abuse) [3]; risky sexual behaviors [4]; and physical injuries that range from relatively minor injuries to disfigurement, permanent disability, life-threatening injuries, and death.

Although IPV affects everyone regardless of age, socioeconomic status, sexual orientation, gender, race, religion, or nationality, research has demonstrated that lesbian, gay, bisexual, transgender, or queer (LGBTQ) individuals experience IPV at rates that are similar to or higher than heterosexual individuals [5,6]. Similarly, ethnic minority women are affected disproportionately by IPV [7], with further disparities noted when individuals are of low socioeconomic status and of foreign-born status [7]. However, the reasons why LGBTQ individuals and ethnic minority women do not seek formal help from health care providers differs. LGBTQ individuals state that they are often reluctant to seek formal help when services tailored to LGBTQ individuals are not available, when health care providers are not sensitive to LGBTQ issues, when they distrust providers, when they fear *coming out* to their provider, and when they believe that the abuse would not be taken seriously [5]. In contrast, perceived discrimination, immigration status, and mistrust of medical professionals are barriers to ethnic minority women reporting or seeking support for IPV [7]. There is strong evidence that persons with a history of IPV have higher health care utilization rates than persons with no history of IPV [8], even if their visit is unrelated to the abuse [9]. In addition, the increased use of health care resources by abuse survivors does not end when IPV ends, but it continues for up to 16 years after the abuse ends [10].

More than 75% of practicing nurse practitioners provide care in primary care settings [11] and are in the unique position to play a vital role in identifying and evaluating IPV, providing assistance and support to victims, and linking victims to specialized support services [12,13]. Unfortunately, the extant literature indicates that many nursing professionals exhibit poor levels of content knowledge and competence that negatively influence their ability to broach the topic with patients and respond in an appropriate manner [14-16]. Although the US Preventive Services Task Force recommends that women of childbearing age be screened for IPV by their clinician at each visit and that interventions or referrals should be provided as indicated by screening results [17], clinicians identified lack of IPV knowledge and confidence as barriers to screening [18,19].

An effective method by which nurses’ skills and knowledge about IPV screening and intervention can be improved is to educate them on the dynamics of IPV and the importance of intervention and appropriate care [20,21]. IPV is included to some degree in all prelicensure registered nursing programs in the United States to prepare students for their National Council Licensure Examination. The extent of education, method of education (didactic, simulation, and Web-based modules), and where in the curriculum (psychiatry/mental health and foundations of nursing) it is included are not standardized, resulting in students enrolled in postmasters nurse practitioner certificate programs having varying degrees of IPV knowledge.

The blended learning approach that is becoming an increasingly widespread approach in higher education institutions might provide a useful way to augment face-to-face nursing education regarding IPV. In addition to improving learning satisfaction, communication self-efficacies [22], and knowledge [23,24], blended learning environments also promote flexible student learning [25]; learner autonomy [26]; and self-reported reasoning, decision making, and metacognition [24]. Completion of Web-based education on falls risk assessment resulted in undergraduate nursing students’ (NSs) increased knowledge and report of increased self-efficacy when performing a falls risk assessment on an older adult [27], suggesting the effectiveness of Web-based education on NSs’ ability to translate theory into practice.

In summary, there is good evidence to support the integration of Web-based modalities to augment face-to-face nursing education. However, successful implementation of a blended learning system requires careful thought about how the course content will be delivered [28,29], how instructors and students can successfully use the technology, and how the system will support different learners within various learning contexts. To ensure that the system can achieve these goals, electronic learning (e-learning) products should undergo evaluation to identify usability problems and measure product usability. A pedagogical usability framework integrates aspects of standard usability testing (ie, effectiveness, efficiency, and satisfaction) [30], while also addressing the usability features specific to the design of e-learning systems (eg, learner control, motivation, flexibility, and feedback). Thus, usability in the context of e-learning systems concerns whether the elements and content of the system enable students to achieve learning goals with positive learning experiences [31]. In this study, we conducted a pedagogical usability testing [32] of the mPOWERED system for comprehensive health care provider–focused IPV education (Health Equity Institute) and to determine the acceptance of the system and its features in United States–based nurse educators (NEs) and NSs.

### The mPOWERED System

**Figure 1:**
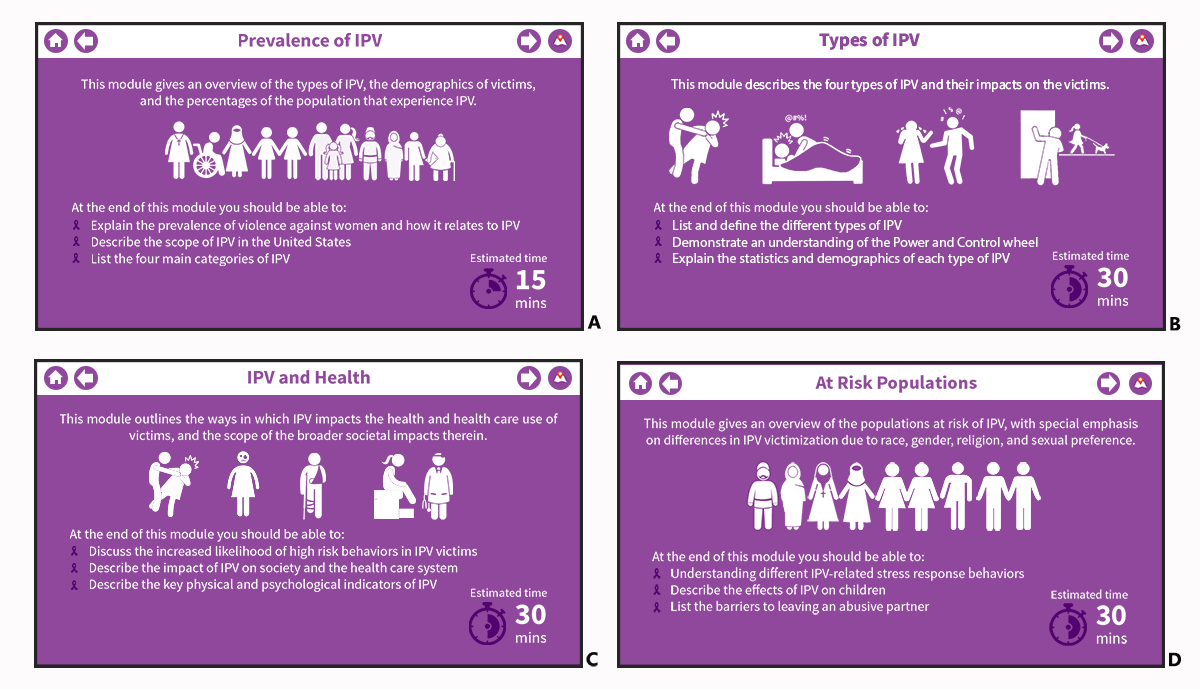
Screenshot of the 4 mPOWERED system modules: (A) Prevalence of Intimate Partner Violence (IPV), (B) Types of IPV, (C) IPV and Health, and (D) At-Risk Populations.

Existing literature indicates that interventions and treatments focused on reducing IPV must address myths and misconceptions and provide IPV-related training that is based on up-to-date and geographically and culturally contextualized empirical evidence. Using the most current empirical evidence and input from NEs and IPV health care providers, the current version of the mPOWERED system comprises 4 modules (Figure 1).

The first module (*Prevalence of IPV*) focuses on definitions of abuse and violence, distinguishing between IPV myths and facts, and acknowledges preexisting values, attitudes, beliefs, and experience held by nurses and how these influence a nurses’ interaction with patients. The second module (*Types of IPV*) defines the 4 types of IPV (ie, physical violence, sexual violence, psychological violence, and stalking), explains the statistics and demographics of each type of IPV, and presents the multicultural power and control wheel [32] to help the NSs and NEs understand how an abuser uses power and control to establish and maintain control over a partner. The third module (*IPV and Health*) discusses the increased likelihood of stress-response behaviors in IPV victims and describes the impact of IPV on society and the health care system as well as the key physical and psychological indicators of IPV. The last module (*At-Risk Populations*) introduces vulnerable populations who are at higher risk of IPV victimization (eg, LGBTQ people and ethnic minorities), describes the effects of IPV on children, and lists the barriers to leaving an abusive partner.

The graphical components of the mPOWERED system were designed using Adobe Creative Suite in accordance with Mayer’s principles of multimedia design [33]. InVision prototyping software was used to transform the static design into a clickable and interactive prototype. Participants completed the usability testing on the interactive InVision prototype using a 7-inch Android tablet (Samsung Galaxy Tab A), where they were able to click on custom-built hotspots to navigate the prototype.

## Methods

### Sampling and Recruitment

To gain the necessary insight into the needs, requirements, and expectations of the mPOWERED system, semistructured interviews were held with NEs and NSs from the San Francisco Bay Area. To be eligible to participate, NEs needed to have experience of working in clinical settings and sufficient experience of teaching in nurse education programs. NSs were eligible to participate if they were currently enrolled in a nurse practitioner certificate program at a US university. Research from the field of usability testing indicates that 80% of usability issues that can be identified in wider implementation will be identified with samples as small as 4 to 5 participants [28]. With that in mind, we used purposive sampling to recruit a maximum variation sample of *key informant* NEs and NSs with nursing experience and clinical practice, as this method provides rich insights into the research topic. Before data collection, ethics approval was granted by the San Francisco State University Institutional Review Board.

### Data Collection and Analysis

Method triangulation was used to increase the validity of findings and gain a more comprehensive understanding of pedagogical usability issues [34]. Specifically, the usability of the mPOWERED system was evaluated utilizing cognitive walkthrough using a concurrent think-aloud method [35], semistructured one-on-one interviews, and a questionnaire with Likert scale and open-ended questions.

At the start of testing, participants were familiarized with the mPOWERED system for approximately 5 min. The participants then performed 4 tasks of varying levels of complexity that covered the full range of functions offered by the mPOWERED system: (1) log in to the system and read through the introduction to the system, (2) work through the first module (*Prevalence of IPV*) and complete the interactive quiz, (3) access the system settings and go to the help center, and (4) work through the fourth module (*At Risk Populations*) and periodically check your progress via the roadmap.

As participants were navigating their way through these tasks, they were encouraged to vocalize anything that crosses their mind (eg, thoughts about any aspect of the system that they liked or disliked, found easy or difficult to understand, or found confusing or contradictory and when they encountered technical difficulties). In addition, participants could receive help from the moderator if they encountered problems or could not manage to go further in the system.

Immediately following usability testing, participants were asked a series of semistructured questions on the perceived usefulness, ease of use, and clarity of information of each system module as well as their experiences and perceptions of the mPOWERED system. The goal of this process was to obtain the participants’ immediate interpretation of a given task scenario and system design and to facilitate the elaboration of usability issues and increasing insight and design suggestions [36]. During this phase, participants were encouraged to discuss the situations where they encountered problems or expressed concerns and then discuss the possible causes of the situation or possible design changes that could be implemented to address the identified issues.

After testing, participants completed a Web-based survey implemented using Qualtrics Web-based survey software that contained questions about the perceived usability of the system, participant demographics (age, gender, position title, and prior experience with IPV), and technology use. The perceived usability of the system was evaluated by the 39-item e-learning usability questionnaire [37] and open-ended questions. The e-learning usability questionnaire measures respondents’ perception regarding the usability of e-learning apps along 7 dimensions of usability (content, learning and support, visual design, navigation, accessibility, interactivity, and self-assessment and learning). The statements were rated on a 5-point Likert scale, with statements ranging from strongly disagree (1) to strongly agree (5), and open-ended questions related to usability. The e-learning usability questionnaire has demonstrated high Cronbach alpha values (>.94), indicating an excellent internal consistency that is adequate for usability testing. Open-ended questions provided participants with the opportunity to write freely about mPOWERED features they found the most/least useful and the barriers to using the system to augment IPV education. Example questions included “What are your opinions (visual design, navigation, etc) of the mPOWERED system” and “What problems did you experience while using the mPOWERED system.”

To evaluate respondent’s prior experience with IPV, we first provided the Centers for Disease Control and Prevention’s definition of IPV and then asked “With this definition in mind, please mark all of the following categories in which (to your knowledge) intimate partner violence has occurred, or is currently occurring,” with the following categories: (1) in your personal experience, (2) immediate family members, (3) close friends, (4) extended family and friends, (5) coworkers or clientele in a work environment, and (6) no instances of IPV. If the participants responded in the affirmative to categories (1) to (6), they answered a follow-up Likert scale question regarding the cumulative number of instances of IPV that occurred in each selected category. Response options for this question were (1) never, (2) once, (3) more than once, (4) a few times, and (5) many times.

With prior approval of the participants, the interviews and focus groups were video recorded using Morae usability and analytics software. All participant and moderator comments along with feedback were independently transcribed using qualitative content analysis [38,39] by 2 members of the research team. Themes generated from the content analysis were mapped to usability heuristics for e-learning systems [30,37]. The usability heuristics are (1) content, (2) consistency and mapping, (3) visual design, (4) navigational fidelity, (5) accessibility, (6) interactivity, (7) self-assessment and learnability, (8) match with the curriculum, and (9) understandable and meaningful symbolic representations. Interresearcher consistency was evaluated by exchanging coded sections of the transcripts, with initial codes and themes reviewed for a second time. When disagreements arose, the researcher made comments on the codes/themes and suggested changes to the coding classification. Disagreements around themes were resolved by an in-person research team discussion until consensus was reached and a final theme was agreed upon [39].

## Results

### Participants

The participants’ characteristics are reported in Table 1. Overall, 7 NEs from San Francisco State University and 18 NSs participated in this study. The mean age of NEs was 58.4 (SD 10.6) years, with 1 individual self-identifying as a man, 5 individuals self-identifying as a woman, and 1 individual self-identifying as genderqueer. The mean age of NSs was 37.0 (SD 6.6) years, with most of the students self-identifying as female (3 men and 16 women). The respondents self-reported a high level of computer use and literacy and diverse personal experiences with IPV.

**Table 1:** Participants’ demographics, clinical and teaching experience, electronic learning, and intimate partner violence experience.

Characteristics		Nurse educators	Nursing students	Overall
Age (years), mean (SD)		58.4 (11)	37 (7)	47.7 (15)
**Gender, n (%)**				
	Male	1 (14)	3 (16)	4 (15)
	Female	5 (71)	16 (84)	21 (81)
	Genderqueer	1 (14)	0 (0)	1 (4)
**Ethnicity, n (%)**				
	Caucasian	2 (29)	5 (28)	7 (28)
	Black/African American	1 (14)	—^a^	1 (4)
	Asian/Asian American	3 (43)	9 (50)	12 (48)
	Hispanic/Latino	1 (14)	2 (11)	3 (12)
	Hawaiian or Pacific Islander	—	1 (6)	1 (4)
	Other	—	1 (6)	1 (4)
**Clinical experience (years), n (%)**				
	<1	—	4 (22)	4 (16)
	1-5	—	4 (22)	4 (16)
	5-10	1 (14)	6 (33)	7 (28)
	>10	7 (100)	4 (22)	11 (44)
Teaching experience (years), mean (SD)		12 (3)	N/A^b^	—
**Mobile devices to facilitate learning, n (%)**				
	Daily	4 (57)	13 (72)	17 (68)
	Twice weekly	2 (29)	3 (17)	5 (20)
	Twice monthly	1 (14)	2 (11)	3 (12)
**IPV** ^c^ **experience, n (%)**				
	No instances of IPV	—	2 (11)	2 (8)
	Current or past relationships	2 (29)	6 (33)	8 (32)
	Immediate family members	1 (14)	8 (44)	9 (36)
	Close friends	2 (29)	6 (33)	8 (32)
	Coworkers or clientele in a work environment	3 (43)	6 (33)	9 (36)

^a^No respondents.

^b^N/A: not applicable.

^c^IPV: intimate partner violence.

### System Usability

Overall, 3 themes emerged from the data to describe NEs’ and NSs’ views about the usability of the mPOWERED system. These were (1) ease of use, (2) usefulness, and (3) aesthetics. Selected ad verbatim quotations from the interviews are presented to illustrate these themes.

#### Ease of Use

This theme included the subthemes content presentation and navigational fidelity. The general opinion among respondents was that IPV content was presented in small understandable chunks (NE mean 4.86; NS mean 4.71) and in a way that supports learning (NE mean 4.86; NS mean 4.65):

I like that you guys broke it down into modules, like small little chunks. And that you can do the quiz and see the answers right away. Yeah, full of rich information.

NE3

I would say this was put forth very well, because you don’t overwhelm in a module. I have modules for nursing where there’s 27 topics just in one, in one go- so it’s really hard to remember some of that stuff. But here, you’ve outline- to me the most important things that you’ve highlighted is the prevalence of IPV, who are the victims- so we know that it’s pretty much all ages, um all socioeconomic um demographics. And you’ve included statistics as well, and the types of IPV. So I think that it’s presented very straightforward, not too much.

NS18

An important facet of e-learning and blended learning is enabling the learner to control the amount of material they consume as well as when they learn the material. Usability testing indicated that most respondents found that the mPOWERED system enabled them to control the pace of learning (NE mean 4.43; NS mean 4.71), with 1 NS reporting:

I like that this whole system is very simple and straight forward. It makes it easier. I think any students who are using this will enjoy it, and it’s not really a looong, dry, or boring lecture. It’s actually quite interesting. It keeps it very simple to the point.

NS5

Participants felt that content was presented in a consistent manner (NE mean 4.71; NS mean 4.59) but mentioned that integrating audio narration into the system would emphasize the organization of key points in the material and reduce the overall cognitive demands placed on the user:

There is a lot of statistics and a lot of information, like minute information. So I would say having this presentation narrated it’s gonna be helpful. And forcing the reader to stop and look at the different types of x, y, and z.

NE6

#### Usefulness

Aspects of usefulness included consistency with the curriculum and system interactivity. Overall, both NEs and NSs expressed liking the mPOWERED system, finding that the content was congruent with the manner in which IPV content is taught in the classroom setting (NE mean 4.86; NS mean 4.65):

I thought this was an excellent way to present IPV to students. It was informational, provided interaction with the module, and user friendly.

NS5

Participants were of the view that the mPOWERED system could augment classroom learning of IPV (NE mean 4.86; NS mean 4.45), with all NEs stating that the system added flexibility in the teaching and learning environment, and would allow them to have deeper and richer teacher-student interactions during in-person classroom meetings:

I would use it to do, like before going to class for the lecture. Cos [Because] that will have them primed to start conversations and share stories about IPV, whether they know about it, or heard about it, or seen it, in a clinical setting.

NE4

Participants also stated that the mPOWERED system engaged the user (NE mean 4.71; NS mean 4.61) and motivated them to learn about IPV (NE mean 4.43; NS mean 4.71). This was especially true for the short multiple-choice quizzes at the end of each module. Both NSs and NEs unanimously felt that quizzes encouraged active learning and especially appreciated that the system provided adaptive feedback on students’ answers after each question:

I like the quiz because it’s interactive and you click on it and it gives you the answer right away. As opposed to answering the questions and then having the answers at the end. So I like this format. So keep that definitely.

NE3

**Figure 2:**
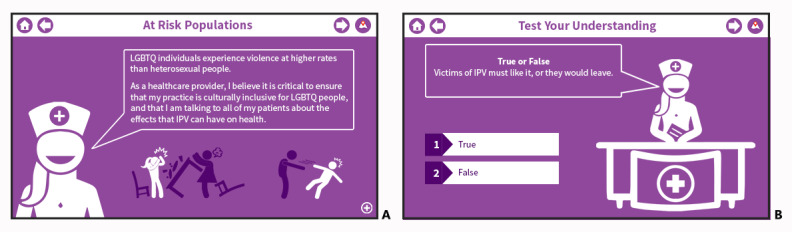
Exemplar screenshots of a (A) health care provider slide and (B) interactive quiz question.

One facet of the mPOWERED system that NSs particularly liked was the health care provider slides (see Figure 2) that students believed helped reinforce the content of the mPOWERED system:

My favorite thing is the doctor/nurse speaking to you at various junctions of the program. This strengthens the messages.

NS15

Love the use of “I,” and the communicative approach. [...] She’s telling you this. That’s what I first thought at the beginning, I really like that you’ve kept it going throughout.

NS18

In contrast, many NEs did not like the health care provider slides, with respondents expressing a dislike for the first-person perspective:

It was distracting and it made me think “oh well ok, they are trying to plant the seed in my head that I’m supposed to think this way.” I see it as a distraction and the way I perceived it in my head is that they were trying to subconsciously make me think that way.

NE4

In general, participants were satisfied with the mPOWERED system, finding that it improved their understanding of the content material. However, both NSs and NEs stated that the system content should be expanded to include information regarding identifying and evaluating IPV and how to offer first-line support for IPV:

Well this just says how it impacts, it doesn’t tell me what I can do.

NE5

This tells me about it [IPV], but not what we do about it. I’d want to know what to do.

NS18

#### Aesthetics

The theme aesthetics explored aspects including visual design and understandable and meaningful symbolic representations. First, there was a high level of satisfaction among the NSs and NEs regarding the balance between text and graphics on each slide (NE mean 4.78; NS mean 4.82), with participants reporting that the IPV-related icons were easy to interpret (NE mean 4.57; NS mean 4.18):

I think the size of the icons are good here, they are clear as to what they represent...These as well. So without the words I could likely put together a lot of>what’s going on here.

NS4

The icons are good. They’re pretty straightforward, in terms of like if you took away the titles you could probably deduce what they’re meaning.

NE5

In addition to comments about the symbolic representations, the use of icons to illustrate IPV-related scenarios was well received by participants and conveyed the seriousness of the issue in a compelling fashion (NE mean 4.71; NS mean 4.18):

I do really like the pictures. Yeah, those are good. I think it shows distress in a sense that, I guess it’s just a matter of visualizing the significance of violence, you know? And kind of seeing what someone is going through in the intimacy of their home and just kind of seeing it as a kind of significant thing. It has this emotional thing where you are more connected to the content. You think it could come off as cheesy, or minimizing it, like characters getting beaten up. But I think it helps you just understand the importance of “this is what someone is going through.” And especially in this particular way, and seeing the way the body is contorted, it doesn’t make it so graphic. But it still is kind of like “ohhh”.

NE3

I like the fact that its pictures, and not real people. Cos [Because] that doesn’t sit well with me when I watch actors screaming at each other.

NE4

**Figure 3:**
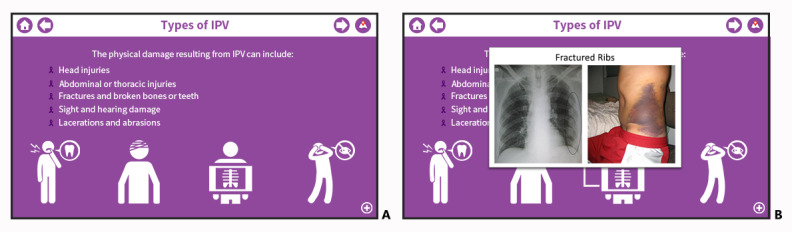
Screenshots of (A) initial and (B) modified intimate partner violence (IPV) and health module slides revised to medical images and radiologic findings of physical injuries associated with IPV.

That said, others suggested the use of real graphics to improve the graphical depiction of scenarios. For example, 1 NS recommended adding actual photos to the system to complement the existing icons (see Figure 3):

You know, actually, for healthcare professionals or people that you want to show this information to I think real photos would probably really hit home. In terms of like drawing them into the emotional side, the magnitude of what’s going on.

NS18

In general, participants felt that the system avoided reinforcing negative stereotypes (NE mean 4.43; NS mean 4.59), with icons portraying diverse demographics and numerous situations in which IPV can manifest itself. However, 1 NE with expertise in LGBT issues felt that some of the icons were too stereotypical and outdated:

Here’s another thing to think about. Its binary, it’s always women and men. I think that’s something that we are having to look at when we are discussing things now. Is it important to say women and men, or people who identify as women, or people who identify with me. Because I would say this number shoots up when it is a transwoman. And actually a transman. Actually they are really vulnerable in relationships.

NE2

In general, this is men on women, which I think for nurses I think that they should understand that there are different types of violence. Like slashing his tire wheels, cutting up his clothes that are in his closet...I would want to see it if we are teaching all sorts of health professionals for it to be more broader.

NE2

## Discussion

### Principal Findings

The aim of this study was to conduct usability testing of the mPOWERED system of IPV education and to determine the acceptance of the system and its features among NEs and NSs. Overall, the mPOWERED system was well accepted by most respondents as an alternative teaching/learning modality. Participants’ comments suggest that the ease of navigating the system and amount of content were appreciated and that the e-learning system provides a user-friendly, visually appealing means that can alleviate the difficulty of adding IPV education in already content-heavy nursing program curriculums [40].

Positive user experience is of prime importance to educators and educational systems. As adult learners, students are more engaged when learning directly relates to their professional career [41]. Framing information from the perspective of a health care professional, and including audio case studies in the mPOWERED system, provides context to the IPV education and its relevance to the participant’s role in the nursing profession. The inclusion of statistical information with direct links to the empirical evidence enables educators to easily update content, thus ensuring that the users can directly access current information. The ability to review information and receive immediate feedback on each test question were positive aspects of the mPOWERED system that participants specifically commented on and felt meet the needs of the self-directed adult learners.

The usability of e-learning software products is a key characteristic to achieve the acceptance of academic users [42] and health care professionals regardless of their background, experience, or orientation [43]. The usability testing and semistructured interviews uncovered aspects of the mPOWERED system that can be improved and expanded in future developments. First, our original intent during icon development was that the icons should be quickly recognized and processed, while at the same time avoiding sensationalizing and trivializing this serious public health issue. Although both NSs and NEs liked the icons and thought that they were easy to interpret, 1 NE with expertise in LGBT issues felt the mPOWERED system could be improved by updating the “too stereotypical and outdated” icons. There is evidence that the interpretation of health-related icons is influenced by interrelating factors (eg, culture and literacy) [44], and as such, we will employ user-centered design methods to evaluate user’s responses to the health information visualization icons when modifying the current modules and developing additional modules of the mPOWERED system.

Second, participants expressed that adding medical images and radiologic findings to the mPOWERED modules would provide users with realistic images that could facilitate the identification and evaluation of IPV. Cognizant of the fact that 25% of women and 10% of men in the United States have been a victim of IPV during their lifetime [45] and that realistic images may trigger extreme emotions, we decided to implement the realistic images via a modal window (ie, a window element that sits on top of an app’s main window) that users could access by clicking on the relevant icons (see Figure 3) rather than having the medical images appear directly in the lesson content pages.

Third, participants commented that the mPOWERED system should include a module providing evidence-based information on how to (1) identify and evaluate IPV and (2) offer first-line support for individuals experiencing IPV. The expressed desire for this specific additional information is consistent with the literature indicating that the lack of education and training poses a significant obstacle to IPV screening and support [18,19], and the next mPOWERED system modules to be developed will focus on first-line responses. This is especially relevant for nurse practitioner students who will be practicing in primary care settings. In addition, our future plans include integrating modules focusing on the root causes of IPV (ie, power and control) using an empowerment and gender equality framework.

This study makes a number of unique contributions to the development of blended learning systems for health care–related topics. Using a purposive sample methodology, we were able to get feedback from participants who would likely use this technology, both from the perspective of the educator and the student. After implementation of suggested changes, we plan to evaluate the pedagogical impact of the mPOWERED IPV education system to improve IPV knowledge, attitudes, and skills in students enrolled in our nurse practitioner program and then expand its use in our prelicensure nursing program. In addition, we will explore the ability of the system to educate other health care professionals (eg, physical therapists, occupational therapists, and physician assistants) on this serious public health topic.

### Conclusions

NEs need to ensure that students are adequately prepared and competent to identify and evaluate IPV, are able to provide adequate assistance and support to victims, and can refer victims to specialized support services. A usability test conducted to explore users’ experiences with the mPOWERED system indicated that the system was useful, usable, and satisfying. Furthermore, participants proposed that the mPOWERED system would be a useful tool to augment traditional education and were keen to see more blended learning in their program curriculum. Both NEs and NSs identified design issues that will provide direction in the next stages of product development. The mPOWERED system has considerable potential to augment traditional classroom learning about health topics such as IPV.

## Abbreviations

e-learning: electronic learning
IPV: intimate partner violence
LGBTQ: lesbian, gay, bisexual, transgender, or queer
NE: nurse educator
NS: nursing student
